# What we know about grief intervention: a bibliometric analysis

**DOI:** 10.3389/fpsyt.2023.1152660

**Published:** 2023-08-04

**Authors:** Jie Li, Yuan Li, Yali Wang, Wuga Jishi, Jinbo Fang

**Affiliations:** ^1^West China Hospital/West China School of Nursing, Sichuan University, Chengdu, China; ^2^Department of Neonatology, West China Second University Hospital, Sichuan University, Chengdu, China; ^3^Department of Nursing, West China Second University Hospital/West China School of Nursing, Sichuan University, Chengdu, China; ^4^Department of Cardiology, West China Hospital/West China School of Nursing, Sichuan University, Chengdu, China

**Keywords:** grief intervention, bereavement, bibliometric analysis, VOSviewer, Web of Science

## Abstract

**Background:**

Grief is a natural and individualized response to different losses, but if grief persists or becomes pathological, professional interventions are required. Grief and corresponding interventions have received increasing attention, as the related concepts have been incorporated into the DSM-5 and ICD-11. Therefore, we conducted a bibliometric analysis to explore the developments in the field of grief intervention research.

**Methods:**

Articles on grief interventions were systematically searched and screened from the Web of Science Core Collection. The retrieved data were analyzed and visualized using VOSviewer and Bibliometrix software for journals, authors, institutions, countries, references, and keywords.

**Results:**

A total of 9,754 articles were included. The number of articles on grief interventions has increased significantly each year since 1990. *Death Studies* was the journal that published the most articles in this field. We identified 25,140 authors contributed to this research area and these authors were from 123 countries and 6,630 institutions. Boelen PA secured the first position in article production, Columbia University emerged as the most productive affiliation and the United States was the foremost leading in grief intervention research. The prevalent keywords utilized in this field comprised bereavement, grief, death, depression, and palliative care.

**Conclusion:**

The quantity of publications regarding grief interventions is increasing. Although most prior studies have focused on mortality, grief, and health, emerging themes such as COVID-19, grief among workers, and disfranchised grief have drawn increasing attention in recent years. Future studies may focus on investigating the complexities and challenges of grief, including its underlying mechanisms and impact on mental well-being.

## 1. Introduction

Grief is a natural and individualized response to different losses (1), which can come in many forms, such as death of a loved one, loss of a job, breakdown of a relationship, and other unexpected events and changes (2). While loss is an inevitable part of life, the experience of grief can often result in emotional distress, suffering, and potentially negative health outcomes (3). When the experience of grief persists or becomes pathological, it can be linked to increased levels of overall grief, depression, and post-traumatic stress disorder (PTSD) (4, 5). Such conditions may even lead to increased mortality rates (6, 7).

Researchers in the field of grief have developed concepts to define pathological or unusual grief, such as complicated grief (CG), prolonged grief disorder (PGD), and persistent complex bereavement disorder (PCBD). These concepts have proven useful in identifying instances of grief that require intervention and have subsequently been incorporated into official diagnostic manuals (8). PCBD and PGD can help identify individuals who are experiencing prolonged and severe symptoms of grief and who may benefit from interventions. The prevalence of PGD in adult bereaved individuals is estimated to be 9.8% (9), and it can be as high as 49% in those who have experienced non-natural or traumatic bereavement (10). While many people are able to manage their grief independently, some individuals may struggle with intense and persistent symptoms of grief that interfere with their daily life and well-being. These symptoms can include intense emotional distress, preoccupation with thoughts or memories of the loss object, cravings for their presence, diminished interest in life, and a decrease in engagement in activities that were previously enjoyable (11). For individuals who are experiencing prolonged and severe symptoms of grief, it is imperative to seek professional grief intervention to facilitate the process of healing and recovery (12).

Grief interventions (GI) encompass a wide range of techniques, strategies, and therapies that aim to support individuals as they navigate the complex emotional landscape of loss. These interventions are designed to alleviate symptoms of grief, depression, and PTSD by providing targeted support and guidance, typically provided by trained health care professionals (13). Various approaches have been developed and the choice of approach depends on the individual’s needs, cultural background, and personal preferences. Some common types of GI include: grief counseling (14, 15), cognitive-behavioral therapy (16–18), family therapy (19, 20), psychotherapy (21), art therapy (22, 23), pharmacological interventions (24), mindfulness and meditation (25), and internet-based GI (13, 26). These interventions can be particularly crucial for those who are at a higher risk of poor grief coping, such as adult bereaved individuals (21), individuals who have experienced the loss of a loved one to suicide (27), women who have had an abortion (28), families who have lost someone to cancer (29), and young people with anxiety and depression symptoms (30). In addition, GI can target a diverse range of populations, including those who have experienced loss due to disasters, illness, death of a loved one, and even pet loss (31–33). It is imperative to acknowledge that grief is an exquisitely personalized journey, and interventions ought to be tailored to meet the unique needs and circumstances of each individual seeking support.

Although there have been numerous studies on various aspects of GI, there is currently a lack of study that provides a comprehensive overview of the literature in this field. Bibliometric analysis is a quantitative method used to analyze and measure published research output in a particular field or discipline. It involves the use of various bibliometric indicators to gather data on the articles, journals, authors, and institutions that contribute to the field (34). By analyzing this data, researchers can gain insights into the research trends, collaborations, and impact of the field (34). Bibliometric analysis can also be used to identify research gaps and potential future directions for the field (34). This study aims to provide a comprehensive overview of the field of GI by conducting a bibliometric analysis using Bibliometrix and VOSviewer. The insights provided by this study is valuable in guiding future studies and practices, and encourage more researchers and practitioners to contribute to the promotion of GI and improve the lives of those affected by grief.

## 2. Methods

### 2.1. Data collection

**Figure 1:**
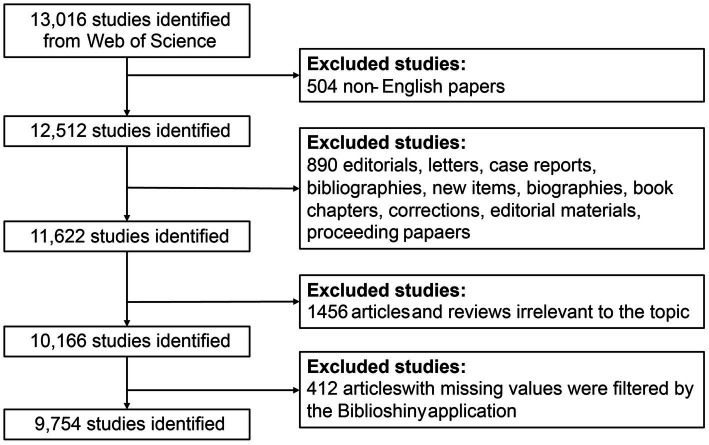
Flow diagram of the literature selection process.

Web of Science (WoS) comprises a vast array of academic journals and literature and is widely regarded as one of the most authoritative and comprehensive databases in multiple disciplines. The data for this bibliometric study was retrieved from the Clarivate Analytics Web of Science Core Collection (WoSCC) database, which is recognized as the optimal data source for bibliometric analysis. We queried the WoSCC online database with the following search string on 9 April 2023: TS = (grief* OR grieving OR mourning* OR bereave*) AND TS = (intervention* OR counsel* OR interview* OR consol* OR therap* OR management OR treatment* OR psychotherap* OR prevention OR program* OR support OR coaching). Only original articles and reviews relevant to GI and published in English from the database’s inception until April 2023 were potentially eligible. Figure 1 presents detailed information on the literature selection process, and a total of 9,754 literature records were included in the analysis. All literature records retrieved from WoSCC were downloaded and exported in the format of Plain Text File with “full record and cited references” as the record content (including titles, keywords, publication dates, origin countries and regions, authors, institutions, published journals, sum of citations, H-index, and other related information) for subsequent visualization and bibliometric analysis.

### 2.2. Data analysis

R studio 4.2.1 (35) and VOSviewer 1.6.18 (36) were used to analyze the retrieved data. The open-source packages Bibliometrix and Biblioshiny running in the R language environment (35) were employed to obtain key bibliometric indicators, including annual scientific output, most productive authors, journals, and institutions, national or regional collaborations, most cited documents, keyword analysis, conceptual structure, and trend analysis of topics. VOSviewer was applied to perform and visualize the co-authorship of authors, institutions, and countries; the co-occurrence of all keywords; and the bibliographic coupling analysis of authors, institutions, countries, and references.

### 2.3. Ethics statement

Ethical approval was not required for the current study, as all data in the bibliometric analysis were downloaded from a public database, and no animals or humans were involved.

## 3. Results

### 3.1. Publication outputs

**Figure 2:**
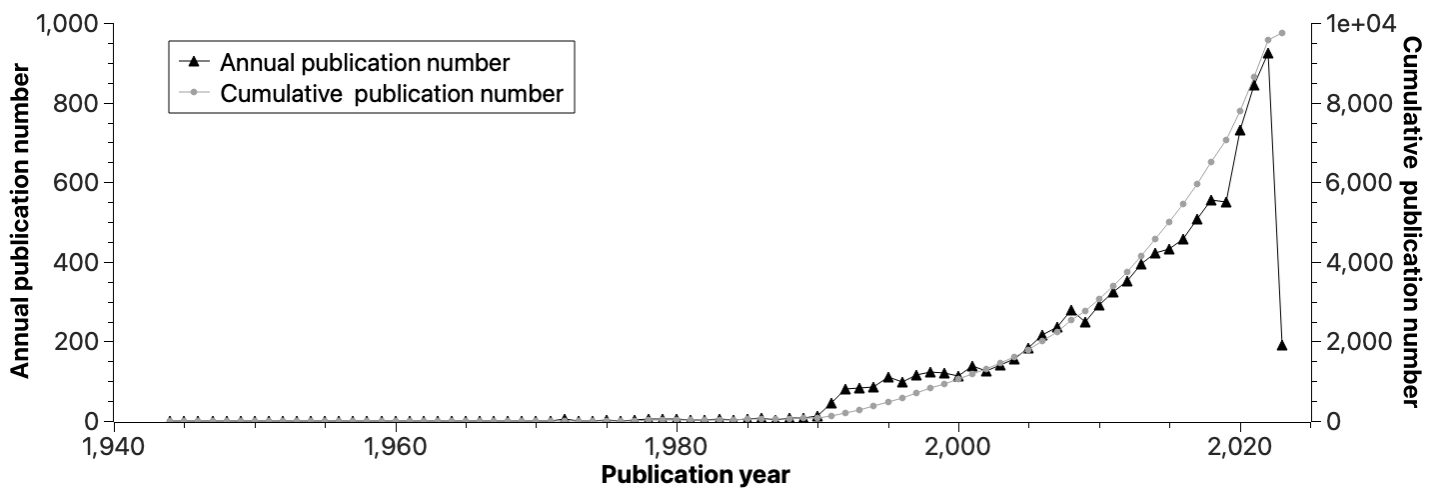
The number of publications by year.

Table 1 gives a summary of the bibliometric data that were used to conduct the bibliometric study. The retrieved literature spanning from 1944 to 2023 were published in 1,670 different journals and written by 25,140 authors. The authors’ international cooperation rate in the field of GI study is 16.49%. As illustrated by Figure 2, the present study uncovered a consistent upward trend in the aggregate count of published papers since 1990 with an annual growth rate of 6.87%. This trend has culminated in a current total of 9,754 published papers, including 8,979 original articles (92.05%) and 775 reviews (7.95%), and the year 2021 stood out with the highest publication volume (*n* = 845).

**Table 1:** Main information of the collected bibliometric data.

Item	Results
Timespan	1944~2023
Journals	1,670
Documents	9,754
Annual growth rate (%)	6.87
Document average age	10
Average citations per document	26.11
References	222,135
Authors	25,140
Institutions	6,630
Authors collaboration	
Single-authored documents	1,439
Co-authors per document	4.14
International co-authorships (%)	16.49
Document types	
Article	8,979
Review	775

### 3.2. Journals

Table 2 lists the top 10 journals that published the largest number of papers related to GI, covering 21.8% (2,128/9,754) of the total number of publications. Specifically, *Death Studies* published the highest number of papers on GI, accounting for 5.88% of the total with 574 publications, and had a higher H-index compared to other journals in the field. The *OMEGA-Journal of Death and Dying* ranked second with 452 publications. Among the top 10 journals, seven had an impact factor (IF) greater than 3.000 in 2021; *Palliative Medicine* and *Journal of Pain and Symptom Management* had the highest impact factor (IF) of 5.713 and 5.576, respectively. It is worth noting that *Journal of Pain and Symptom Management* was the journal with the highest journal citation indicator (JCI) in 2021, with a value of 1.42. In addition, the top 10 journals included six from the United States, followed by three from the United Kingdom.

**Table 2:** Top 10 most relevant journals in grief intervention research: 1944–2023.

Rank	Sources	Articles	IF_2021_	H-index	JCI_2021_	Country
1	Death Studies	574	4.340	56	1.26	United States
2	Omega-Journal of Death and Dying	452	2.062	39	0.62	United States
3	Palliative Medicine	196	5.713	46	1.24	United Kingdom
4	Journal of Palliative Medicine	165	2.947	36	0.76	United States
5	Journal of Pain and Symptom Management	161	5.576	37	1.42	United States
6	Journal of Loss and Trauma	132	4.775	20	1.06	United States
7	American Journal of Hospice and Palliative Medicine	121	2.090	20	0.64	United States
8	BMC Palliative Care	116	3.113	21	1.01	United Kingdom
9	Palliative and Supportive Care	113	3.733	21	1.12	United Kingdom
10	International Journal of Environmental Research and Public Health	98	4.614	14	0.93	Switzerland

IF_2021_, Impact Factor in 2021; JCI_2021_, Journal Citation Indicator in 2021.

### 3.3. Authors

**Figure 3:**
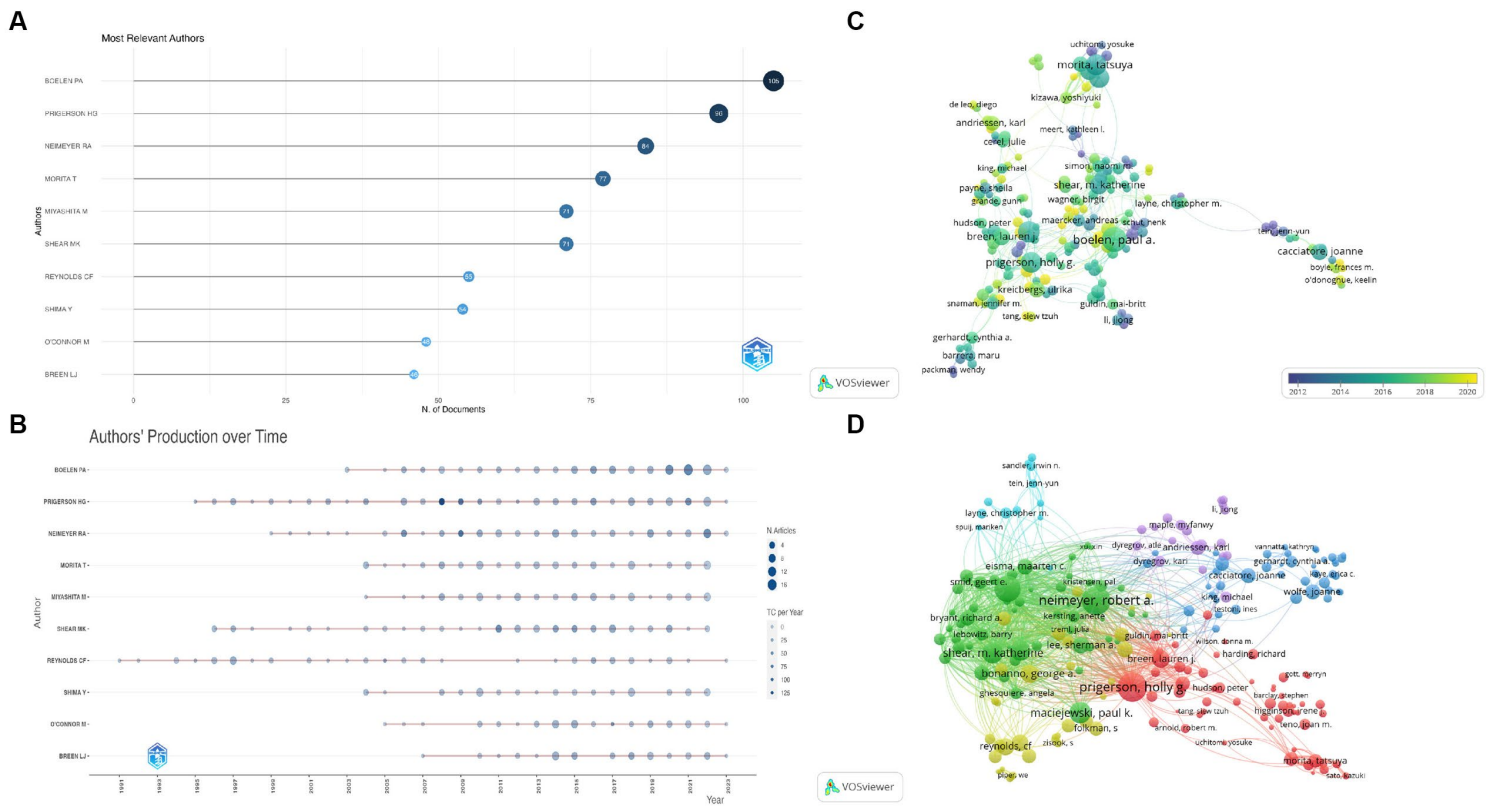
Visualization on main authors in grief intervention research. **(A)** Top 10 most relevant authors. **(B)** Top 10 authors’ production over time (a larger bubble indicates higher productivity in a particular year, whereas a darker bubble indicates a higher number of citations). **(C)** The co-authorship network map of authors (a larger circle indicates a greater quantity of documents produced, while a lighter circle indicates a closer degree of collaboration). **(D)** The bibliographic coupling network of authors (a larger circle indicates a higher Normalized Citation Impact).

Figure 3A shows the top 10 most productive authors in the GI field. A total of 697 articles were written by these authors. Boelen PA ranked first with 105 publications, followed by Prigerson HG with 96 publications and Neimeyer RA with 84 publications. Figure 3B displays the number of publications and citations per year by the top 10 authors over time. The co-authorship network reflects the academic collaboration between two or more authors in a research effort. Figure 3C depicts the co-authorship network in which the unit of analysis is set at the author level and the threshold is set at a minimum of 10 documents per author to construct the network. This means that an author must have published at least 10 articles in the field of GI as either the first author or co-author to be included in the network map. Of the 25,140 authors, 218 met the threshold and connected with other authors in the network. The largest network connection in terms of authorship consisted of 176 entries divided into 19 clusters. The author with the largest node is Boelen PA.

Bibliographic coupling is utilized to delineate relationships among documents, sources, authors, organizations, and countries, which occurs when two distinct works make reference to a shared third work in their respective bibliography (37), and the coupling strength increases as with the number of works shared (38). Figure 3D represents the bibliographic coupling network among authors who have published a minimum of 10 articles pertinent to the GI study. Of the 25,140 authors, 272 met the threshold and were used in the final network. In Figure 3D, the weights have been assigned based on the normalized citation impact (NCI). NCI is a widely used metric for evaluating the scholarly influence of articles or authors (39). By using this metric to assign weights in the bibliographic coupling network analysis, we can identify authors who have made particularly significant contributions to the GI field. Our findings revealed that Prigerson HG holds the most significant weight value in the bibliographic coupling network, followed by Neimeyer RA and Boelen PA.

### 3.4. Institutions

**Figure 4:**
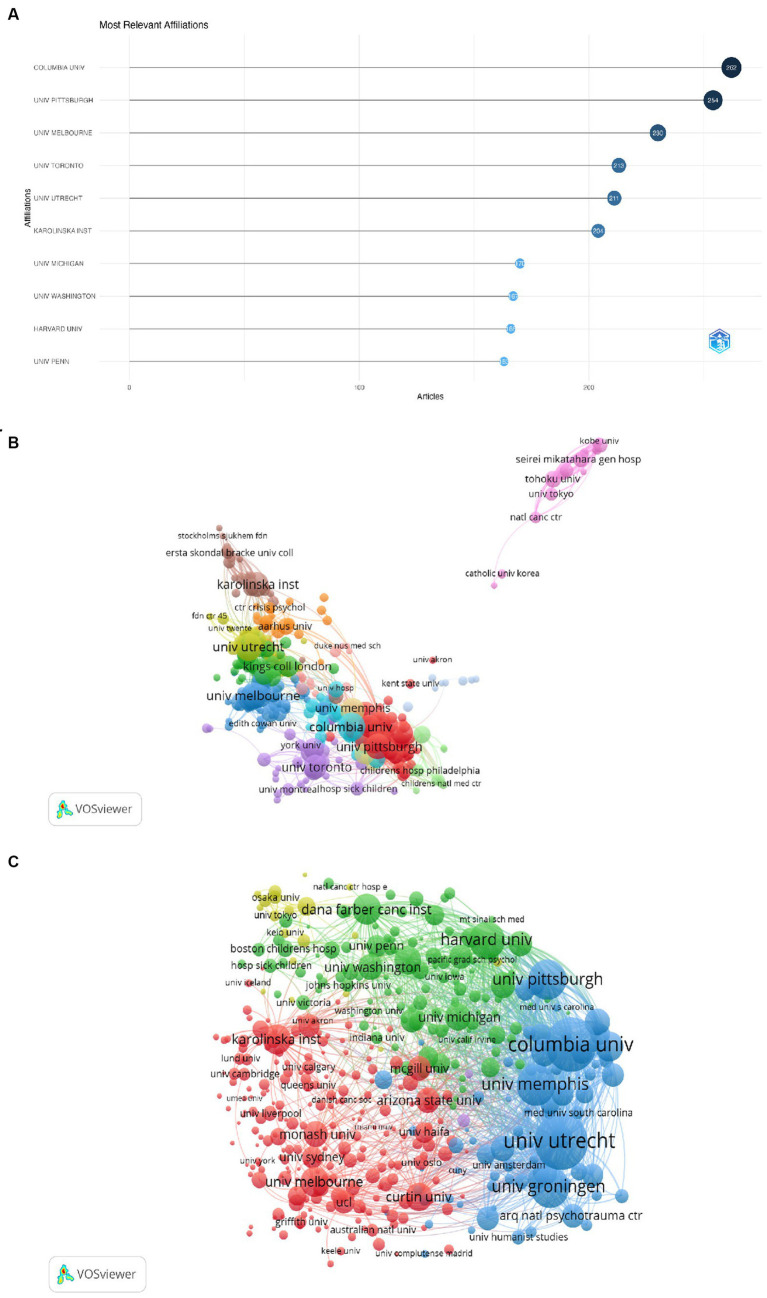
Visualization on main institutions in grief intervention research. **(A)** Top 10 most relevant affiliations. **(B)** The co-authorship network map of institutions. **(C)** The bibliographic coupling network of institutions.

Figure 4A shows the top 10 institutions based on the number of articles published. Columbia University is the top relevant affiliation, with a total of 262 publications by different authors, followed by Utrecht University with 254 publications, and the University of Melbourne with 230 publications in the relevant field. Figure 4B represents the co-author network of main institutions with research publications on GI. Among the 6,630 institutions, 436 reached the threshold of publishing at least 10 documents and entered the network. The largest network connection in terms of institution consisted of 435 entries divided into 19 clusters, with Columbia University serving as the largest node. Figure 4C represents the bibliographic coupling network among main institutions in the GI field. As shown, Utrecht University has the strongest total link strength (a metric evaluating the intensity of connections between nodes within a network) followed by Columbia University and the University of Pittsburgh (40).

### 3.5. Countries

**Figure 5:**
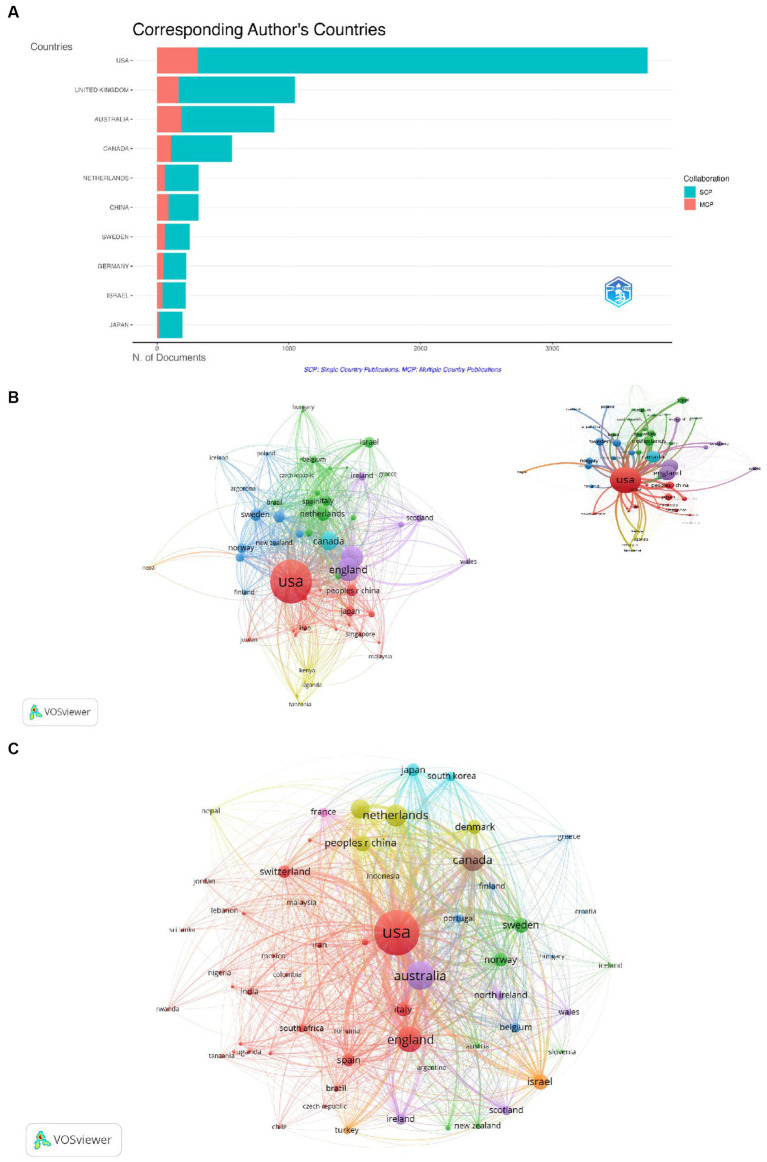
Visualization on main countries in grief intervention research. **(A)** Top 10 most relevant countries (MCP, Multi-country publication; SCP, Single-country publication). **(B)** The co-authorship network map of countries. **(C)** The bibliographic coupling network of countries (the links connecting nodes of different countries indicate collaborations between those countries).

Figure 5A shows the top 10 countries with the highest number of publications. The study results showed that the United States not only had the highest number of published articles, but it also had the highest number of publications authored solely by its own researchers and the most multi-country co-authorship products. In addition, the United Kingdom was the country with the second-highest number of total publications, followed by Australia. Figure 5B shows the co-author network of main countries with research publications on GI. Among the 123 countries/regions, 60 reached the threshold of publishing at least 10 documents and entered the network. The largest network connection in terms of country comprised 60 entries divided into 7 clusters, with the United States serving as the largest node. Figure 5C represents the bibliographic coupling network among the main countries in the GI field. As shown, the United States has the strongest total link strength followed by Australia and England, and evidently, the United States serves as a pivotal node in the network diagram.

### 3.6. Citations

Based on the retrieved data, the earliest known publication related to GI study dated back to 1944, when Lindemann E published an article titled “Symptomatology and management of acute grief.” It is noteworthy that Lindemann’s 1944 article has played a significant role in shaping the GI field and remains one of the most frequently cited documents in the area. Currently, it ranks as the second-most globally cited document. Table 3 gives the top 10 most frequently cited documents, with Fredrickson BL’s 2001 paper titled “The role of positive emotions in positive psychology: the broaden-and-build theory of positive emotions” being the most frequently cited, with a total of 6,456 citations worldwide.

**Table 3:** Top 10 most frequently cited documents in grief intervention research: 1944–2023.

Rank	Paper (Author, year)	Journal	Document	TC	TC/year	Normalized TC
1	Fredrickson BL, 2001	American Psychologist	The role of positive emotions in positive psychology: the broaden-and-build theory of positive emotions	6,456	280.7	67.2
2	Lindemann E, 1944	American Journal of Psychiatry	Symptomatology and management of acute grief	1,883	23.54	1
3	Wright AA, 2008	Journal of the American Medical Association	Associations between end-of-life discussions, patient mental health, medical care near death, and caregiver bereavement adjustment	1,823	113.94	32.51
4	Steinhauser AE, 2000	Journal of the American Medical Association	Factors considered important at the end of life by patients, family, physicians, and other care providers	1,627	67.79	22.28
5	Folkman S, 2000	American Psychologist	Positive affect and the other side of coping	1,341	55.88	18.36
6	Stroebe M, 1999	Death Studies	The dual process model of coping with bereavement: rationale and description	1,111	44.44	22.06
7	Prigerson HG, 2009	PLOS Medicine	Prolonged grief disorder: psychometric validation of criteria proposed for DSM-V and ICD-11	1,107	73.8	30.49
8	Alexopoulos GS, 2005	Lancet	Depression in the elderly	1,046	55.05	19.57
9	Borsboom D, 2017	World Psychiatry	A network theory of mental disorders	1,003	143.29	49.91
10	Stroebe M, 2007	Lancet	Health outcomes of bereavement	997	58.65	17.54

TC, Total citations.

**Figure 6:**
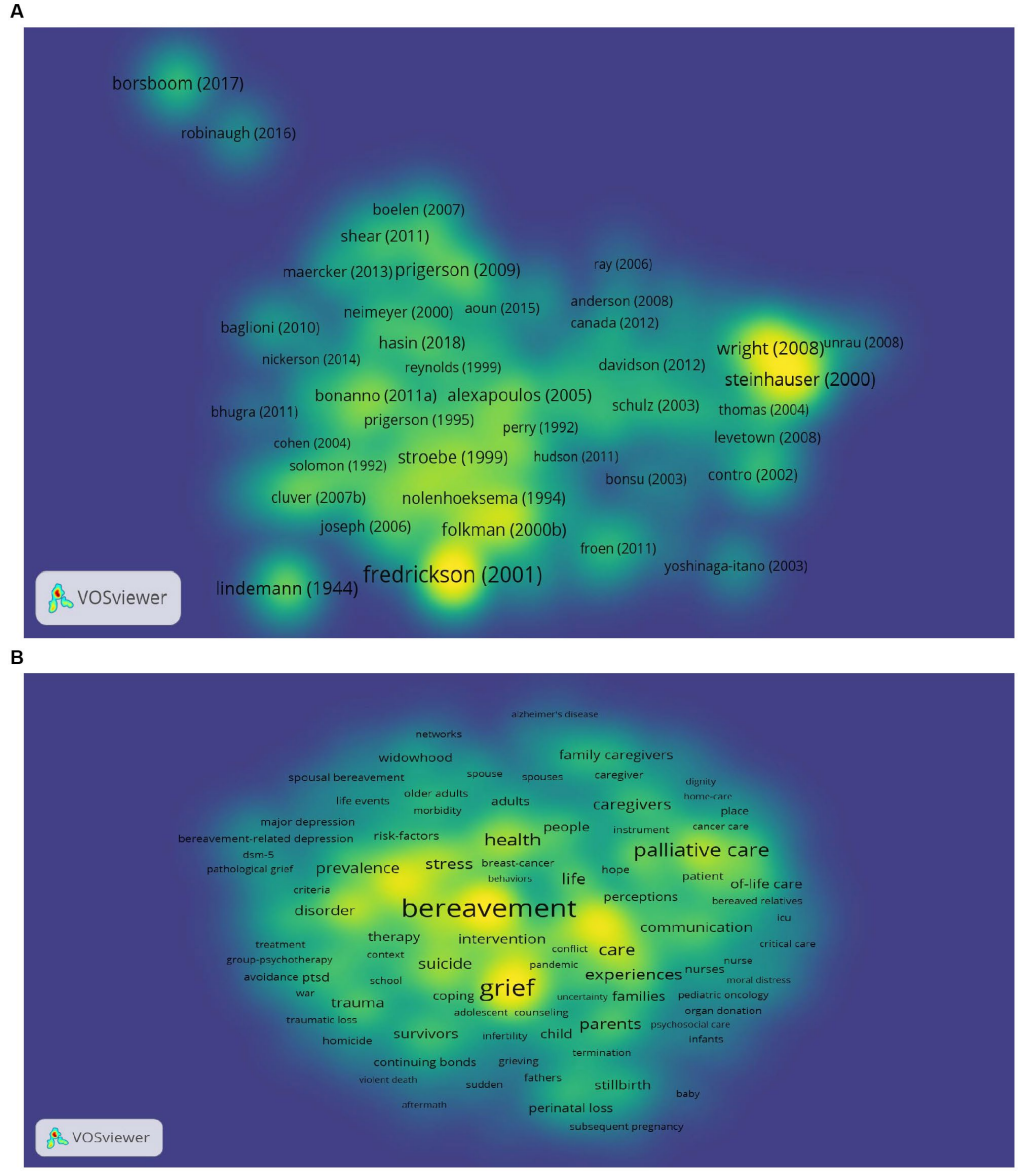
Bibliographic coupling density map (the brighter the color, the greater the corresponding weight.). **(A)** Bibliographic coupling of documents. **(B)** Bibliographic coupling of co-occurrence of keywords.

Given the various advantages of bibliographic coupling analysis, such as its robustness, capacity to detect interdisciplinary relationships and ability to identify connections between publications without any authors in common, combined with the density visualization technique which enables a better understanding of the cited strength of documents, we employed the density map of bibliographic coupling to elucidate the relationships among documents and to identify the most influential ones. Figure 6A shows the bibliographic coupling density map, and the unit of analysis is documents. We set the threshold to include only documents cited at least 100 times and found 391 documents met the threshold, with 386 having the largest set of connections with other papers. The density map revealed denser regions around the documents of Fredrickson BL (2001), Lindemann E (1944), and Wright AA (2008), which is consistent with the findings in Table 3.

### 3.7. Research hotspots and frontiers

**Figure 7:**
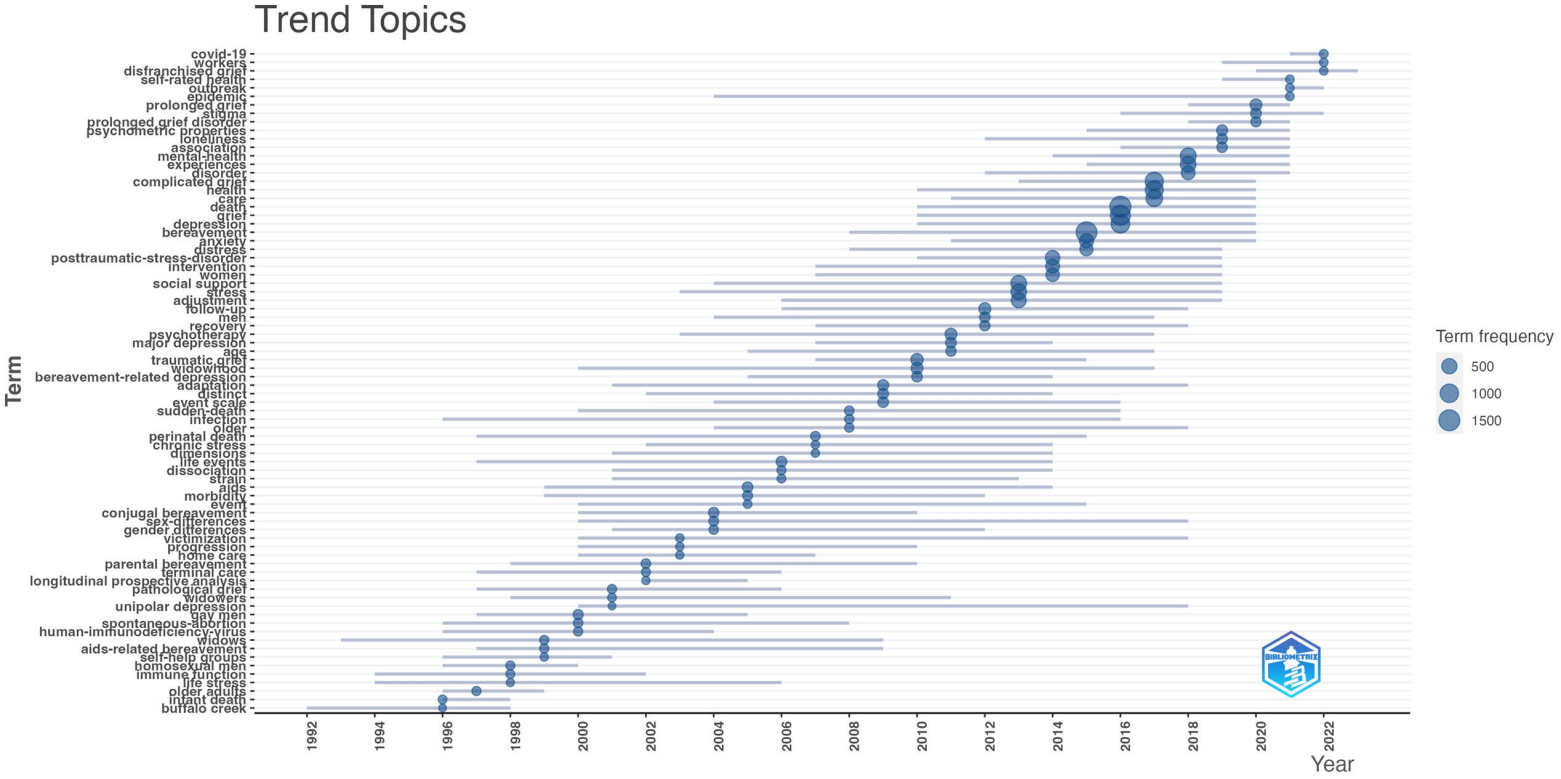
Trending topics: 1944–2023.

Figure 6B shows the co-occurrence analysis of all keywords in the GI domain. The threshold for inclusion was a minimum of 20 occurrences per keyword, with 604 keywords meeting this criterion. The most frequently appearing keywords were bereavement, grief, death, depression, and palliative care. Figure 7 displaying the trending topics in the GI field from 1944 to 2023, as represented by keywords plus, highlighted the research hotspots and frontiers by themes. As shown, “Buffalo Creek” was the first topic appeared in Figure 7, followed by widows, immune function, and life stress. Early research focused on the health and psychological issues of specific social groups or events, such as homosexual men, AIDS, miscarriage, and older adults. As research progressed, it began to address more general health issues, such as chronic stress, bereavement-related depression, adjustment, mental health, and grief, resulting in a broader scope of investigation. Recently, research frontiers of GI study shifted to COVID-19, the well-being of workers, and disfranchised grief and these topics will likely continue to be of great significance in upcoming years.

**Figure 8:**
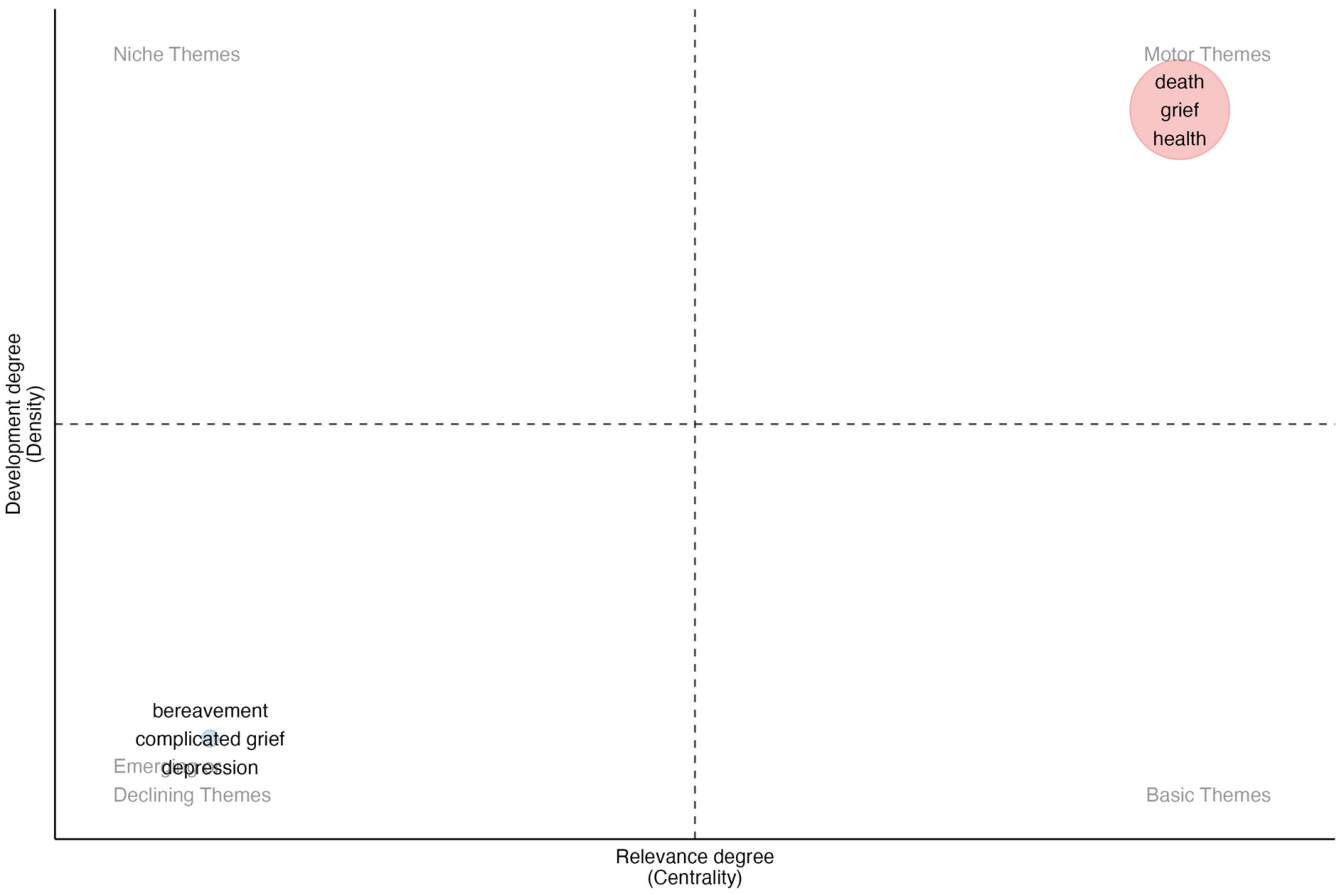
Thematic map for time slice 4 (2020–2023). (Basic Theme: the fundamental and enduring topics in a research field that have been extensively studied and remain relevant over time. Motor Theme: the current and popular research directions that are attracting significant attention. Niche Theme: the specific and relatively less explored topics that are of interest to a limited group of researchers. Emerging/Declining Theme: the newly emerging or declining topics, indicating a shift in research interest.)

Moreover, we conducted a comprehensive analysis of the co-occurrence of all keywords in the past and divided the time period into four distinct time slices. These time slices were as follows: time slice 1 (1944–1999), time slice 2 (2000–2009), time slice 3 (2010–2019), and time slice 4 (2020–2023, up until 9 April 2023). This approach allowed us to perform a thematic map analysis for each time slice, revealing the changing focus areas and main themes of research in the field over time. Thematic maps for each time slice were created to help understand the major themes dividing them into four quadrants based on density (Y-axis) and centrality (X-axis): basic themes, motor themes, niche themes, and emerging or declining themes. The graphical representation of the thematic maps for time slice 1–3 can be found in Supplementary material. Figure 8 shows the thematic map for time slice 4, which is the most recent and relevant to indicate the current research hotspots and frontiers. A total of 2,690 articles were published during time slice 4. Motor themes included death, grief, and health, and there was a shift in the emerging or declining themes, with a focus on bereavement, complicated grief, and depression.

**Figure 9:**
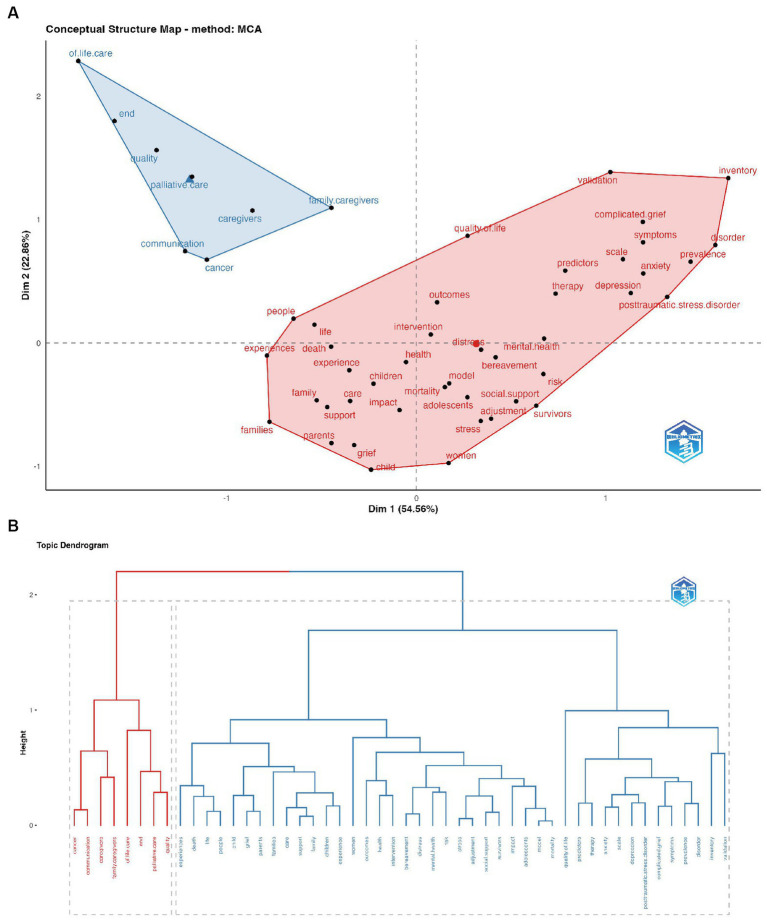
Factorial analysis of author keywords plus using multiple correspondence analysis. **(A)** Conceptual structure map (MCA, multiple corresponding analysis; Dim, dimension). **(B)** Topic dendrogram.

The concept structure function in Bibliometrix uses multivariate correspondence analysis (MCA) to map the conceptual structure of the research field and applies K-means clustering to cluster the literature. The analysis of the conceptual structure uncovers information such as keyword importance and connections, development trends, research frontiers, important research directions, and knowledge gaps in the field. We conducted MCA using the “keyword plus” field, the resulting conceptual structure map is presented in Figure 9A and Figure 9B gives the topic dendrogram map derived from the results of Figure 9A. The factor network resulted in two clusters, whereby each cluster consisted of a minimum of 50 terms. One cluster of keywords was related to palliative care, while the other cluster contained keywords such as death, bereavement, grief, depression, complicated grief, stress, PTSD, anxiety, distress, and so on.

## 4. Discussion

To the best of our knowledge, this is the first bibliometric analysis to explore the development of research related to GI. This study covers articles related to GI published as early as 1944, and the number of publications has been steadily increasing since 1990. Prior to this time, the number of publications produced annually was limited, with the majority focusing on describing or analyzing grief experiences, as well as the development of corresponding theories and treatment methods. The initial stage of the GI study, spanning from 1944 to 1990, can be characterized as embryonic and exploratory. In contrast, the timeframe spanning from 2000 to the present can be characterized by substantial growth, resulting in the publication of 8,816 documents, accounting for 90.4% of the total published to date. Additionally, the rate of international collaboration among authors in the field of GI research is 16.49%, suggesting a need for further efforts to strengthen international cooperation in this area.

The analysis of literature source distribution can help to identify the core journals that publish research on GI, thereby researchers can gain insight into where the most important and influential research is being published. The bibliometric analysis revealed that *Death Studies* was the most influential journal in GI research, with the greatest number of relevant publications in this area and the highest H-index, indicating that this journal has played an essential role in disseminating GI research and published the most cited articles in the field. The fact that 70% of the top 10 most productive journals had an IF higher than 3.000 suggests that the field has been attracting research of relatively high quality. Nevertheless, the absence of top journals with an IF greater than 6.000 underscores the necessity to persist in striving for improvement of the quality of GI research. *Journal of Pain and Symptom Management* stood out as the journal with the highest JCI of 1.42, indicating that the journal held a greater citation impact than the average journal in the field. Furthermore, 60% of the top 10 most productive journals are based in the United States, and no publishers from developed countries feature in this list. Hence, it is imperative to enhance the impact of academic journals in other nations or regions.

The analysis of leading authors, institutions and countries that have contributed to GI research could assist academics in recognizing potential collaborators and understanding existing cooperative relationships. In terms of author analysis, Boelen PA tops the list with a total of 105 publications, and all the top 10 authors are from Europe and the United States. Neimeyer RA appears to have the highest average yearly citation count among these most productive authors. The analysis of research institutions reveals that Columbia University is the most productive and holds the largest node in the cooperation network, indicating that it has the strongest external collaborative relationships in the GI research. Furthermore, Utrecht University exhibits the strongest total link in the bibliographic coupling network, indicating its strong research capabilities, outstanding contributions in this field, and academic reputation and popularity. Regarding the analysis of countries, the United States stands out as the leading country and the hub of a collaborative network. The country’s strong economy and high-quality research institutions may attract researchers from other countries to establish collaborative research relationships (41). Of the top 10 countries with the highest number of publications in GI research, all except China are recognized as developed countries, with six of them ranking in the top 10 countries based on their 2021 GDP. While a country’s psychological development is closely associated with its economic and cultural progress, under-developed countries should also prioritize addressing grief and take action to enhance their psychological well-being. Strengthened international cooperation may be helpful in facilitating such improvements.

The analysis of highly cited articles is essential in locating the most influential publications in the GI research field. Notably, Fredrickson BL’s publication “The role of positive emotions in positive psychology: the broaden-and-build theory of positive emotions” (42) is the most widely cited publication related to GI. In the article, the author proposed the broaden-and-build theory of positive emotions, which suggests that positive emotions can broaden an individual’s momentary thought-action repertoire and construct durable personal resources. The application of the broaden-and-build theory has been widespread in the field of GI research and related areas. For example, by understanding the significance of loss or adopting coping mechanisms for trauma, individuals may attain post-traumatic growth (43). Practicing gratitude exercises has been observed to enhance mental health in the general population amidst the COVID-19 pandemic (44). Internet self-help interventions that focus on cultivating positive thoughts and emotions have also been shown to benefit widowed individuals (45).

Keywords constitute the essence of a paper. Keyword analysis facilitates the summarization of study topics within a particular field and exploration of the current trends and research frontiers (46). This bibliometric study reveals that the most prominent and densely researched topics in the field of GI study are bereavement, grief, death, depression, and palliative care, as evidenced by the keyword co-occurrence density map. Results derived from the trending topics analysis indicated that the calamitous event known as the “Buffalo Creek” disaster marked the origin of GI research and served as the initial focal point that captured widespread interest. Initially, GI research focused on specific social groups or events and their related health and psychological issues. Over time, the scope of research broadened to include more general health issues. In recent years, the COVID-19 pandemic has had a substantial influence on global health, leading to unprecedented levels of loss and grief, affecting millions of people worldwide. Our study findings suggest that the focus of GI research has accordingly shifted to COVID-19, the well-being of workers, and disfranchised grief to account for the most recent challenges.

It is reported by a meta-analysis that the prevalence of grief symptoms and disorders during the COVID-19 pandemic is as high as 45.8% (47). Providing support and effective interventions to help those grieving develop coping mechanisms is crucial for their recovery (48). In recent times technology-based interventions like virtual support groups and online counseling have emerged as viable alternatives for providing grief support amidst the pandemic and in the post pandemic era (44, 45). To address the enduring mental health consequences of grief and loss incorporating further technological advances into grief intervention studies and practices may be necessary. Overall the pandemic highlighted the need to address grief and enabled opportunities for innovation and adaptation in GI research. Amid the pandemic healthcare workers (49) home caregivers (50)and social workers (51) often face various forms of loss which could arise from their clients’ death or grief and the distinct working conditions that affect their grief rights personal health risks and the possibility of unemployment due to being infected. Other ordinary workers and their families also undergo significant grief as per a survey conducted in the United States (52). Our analysis reveals that significant scientific endeavors have been undertaken to address worker grief and improve their overall well-being. Disenfranchised grief denoting the grief experienced by those who suffer a loss but do not or cannot publicly acknowledge mourn or receive social support for it is another topic of interest in current GI research (53). The advent of the COVID-19 outbreak has led to widespread disenfranchised grief resulting from the loss of rights (54). It is critical to acknowledge and tackle this form of grief in diverse situations and devise interventions and supportive measures to help individuals cope with it. According to our bibliographic analysis it appears that in the near future there will be an ongoing trend of GI studies with a focus on COVID-19 worker well-being and disenfranchised grief.

Time slice analysis provides in-depth insights into the development of research topics in the field of GI over time, and the findings from recent years enable us to recognize current research hotspots and frontiers. The results of the study for the time period of 2020–2023 suggest that death, grief, and health are prominent themes in GI research, with a shift toward emerging and declining themes such as bereavement, complicated grief, and depression. This highlights the evolving nature of research in this field, which is now focused on exploring the complexities and challenges of grief and its impact on mental well-being.

Based on the findings of the cluster analysis, GI research can be classified into two categories: GI in palliative care and general GI. In the context of palliative care, the primary objective of GI is to aid patients and their caregivers in dealing with the loss and grief resulting from the disease. While general GI concentrates on helping individuals who are grieving to alleviate their grief experiences, minimize its impact on health, and enhance their recovery. A multidisciplinary team plans the GI based on disease trajectories and the patient’s condition as the illness advances in palliative care. In contrast, general GI primarily focuses on populations who may face adverse outcomes caused by grief, including those who have lost loved ones to non-natural causes and individuals who have trouble adapting to such events.

This study has several limitations that are inherent in bibliometric analyses. Firstly, we only retrieved articles from the WoSCC database and analyzed studies published in English, which may have excluded relevant studies published in other languages or in other databases. Secondly, the data was extracted using machine learning and natural language processing techniques, which may have introduced some biases that have been reported in previous bibliometric studies (55). Additionally, while bibliometric analysis is rigorous, inclusive, and useful for identifying links and clusters, this method may not provide an in-depth analysis of individual articles.

In conclusion, our bibliometric analysis suggests that there has been a consistent growth in research on GI, which has garnered global attention from the academic community. Boelen PA is the most prolific author in this field, while Columbia University has made the most significant academic contributions. The United States is the leading country in GI research, and there is a need for increased international collaboration in this area. Developing countries should also prioritize research on GI. The most cited article in this field is Fredrickson BL’s 2001 paper on the broaden-and-build theory of positive emotions, which provides theoretical guidance for GI studies. While most studies have focused on death, grief, and health, recent years have seen a growing interest in emerging themes such as COVID-19, grief among workers, and disfranchised grief.

## Author contributions

JL, JF, and YL: study design. JL and WJ: data collection, analysis, and interpretation. WJ and YL: writing—original draft preparation. YL, YW, and JF: writing—review and editing. JF and YW: supervision. All authors contributed to the article and approved the submitted version.

## Conflict of interest

The authors declare that the research was conducted in the absence of any commercial or financial relationships that could be construed as a potential conflict of interest.

## Publisher’s note

All claims expressed in this article are solely those of the authors and do not necessarily represent those of their affiliated organizations, or those of the publisher, the editors and the reviewers. Any product that may be evaluated in this article, or claim that may be made by its manufacturer, is not guaranteed or endorsed by the publisher.
