# Supplementary material for: What we know about grief intervention: a bibliometric analysis
Source: Front Psychiatry. 2023 Aug 4;14:1152660. doi: 10.3389/fpsyt.2023.1152660 (PMC10442158; doi:10.3389/fpsyt.2023.1152660)
Supplement: Supplementary file 1 [file Data_Sheet_1.docx]

Supplementary Material

What We Know About Grief Intervention: A Bibliometric Analysis

Jie Li^1†^, Yuan Li^2,3†^, Yali Wang^1,4^, Wuga Jishi^1^, Jinbo Fang^1*^

^1^West China Hospital, Sichuan University/West China School of Nursing, Sichuan University, Chengdu, China

^2^Department of Neonatology, West China Second University Hospital, Sichuan University, Chengdu, China

^3^Department of Nursing, West China Second University Hospital, Sichuan University/West China School of Nursing, Sichuan University, Chengdu, China

^4^ Department of Cardiology, West China Hospital, Sichuan University/West China School of Nursing, Sichuan University, Chengdu, China

^†^ These authors share first authorship.

*** Correspondence:**Jinbo Fang
fangjinbo1107@163.com

# Supplementary data

Figure 1 displays the thematic evolution across the four-time slices from 1944 to 2023. Figure 2 Topic maps were created to understand the quadrants that divide them into four topics: fundamental, automotive, niche, emerging, or declining (again, this parameter was set to Research Keyword Plus). The basic theme represents the classic or main theory or method of this time period, and the motor theme represents the rising hot topic of this time period, which requires our close attention; the niche theme is a niche theme of this time period, but it also needs attention, Emerging themes or declining themes represent emerging themes that are gradually rising in this field, of course, they may also be gradually declining themes, and the specific judgment should be determined according to the text environment.

**Time slice 1 (1944-1999)**

During this time slice, a total of 938 articles were published, as shown in Figure 2(A).

Base themes: The identified basic themes are death/grief/support, bereavement/stress/social support, stillbirth/neonatal death/follow-up, gay men/aids-related bereavement homosexual men.

Motor themes: The identified motor themes are widowhood/mortality/mental health.

Program/anxiety/attachment is located at the intersection of the Basic Themes and Motor Themes quadrants, reflecting the uncertainty and complexity of data analysis. It may also represent their traditional foundation and involvement in new development trends during this time period, which may indicate a high level of innovation and foresight.

Niche themes: The definitive Niche themes are hospice/terminal care/satisfaction, trial/efficacy, patterns, sample/home, and decisions/patient.

Emerging or declining themes: attitude.

The topic of "Aids/men/needs" is located at the intersection of the Basic themes and the Emerging or declining themes quadrants, which may indicate that this theme has a strong traditional foundation during this time period, but also involves some new development trends or changes that require further analysis.

**Time slice 2 (2000-2009)**

During this time slice, a total of 1,838 articles were published, as shown in Figure 2(B).

Base themes: depression/complicated grief/symtoms, bereavement/death/grief.

Niche themes: palliative care

**Time slice 3 (2010-2019)**

During this time slice, a total of 4,288 articles were published, as shown in Figure 2(C).

Base themes: death/grief/health

Bereavement/complicated grief/depression is located at the intersection of Motor themes and Niche themes in the thematic map, it may suggest that this theme has a high level of research activity and has already gained significant attention in the field, but at the same time, it also has a specific and narrow research focus. This intersection indicates that this theme has the potential for continued research and development, but may require further exploration of new directions, methods, and applications. Motor themes: none

Palliative care/end/experiences theme circle is located at the intersection of Niche themes and Emerging or declining themes in the thematic map, it may indicate that this theme has some uniqueness or innovativeness in the research field, while there are also some new development trends or decline trends that need to be further studied and analyzed.

# Supplementary figures


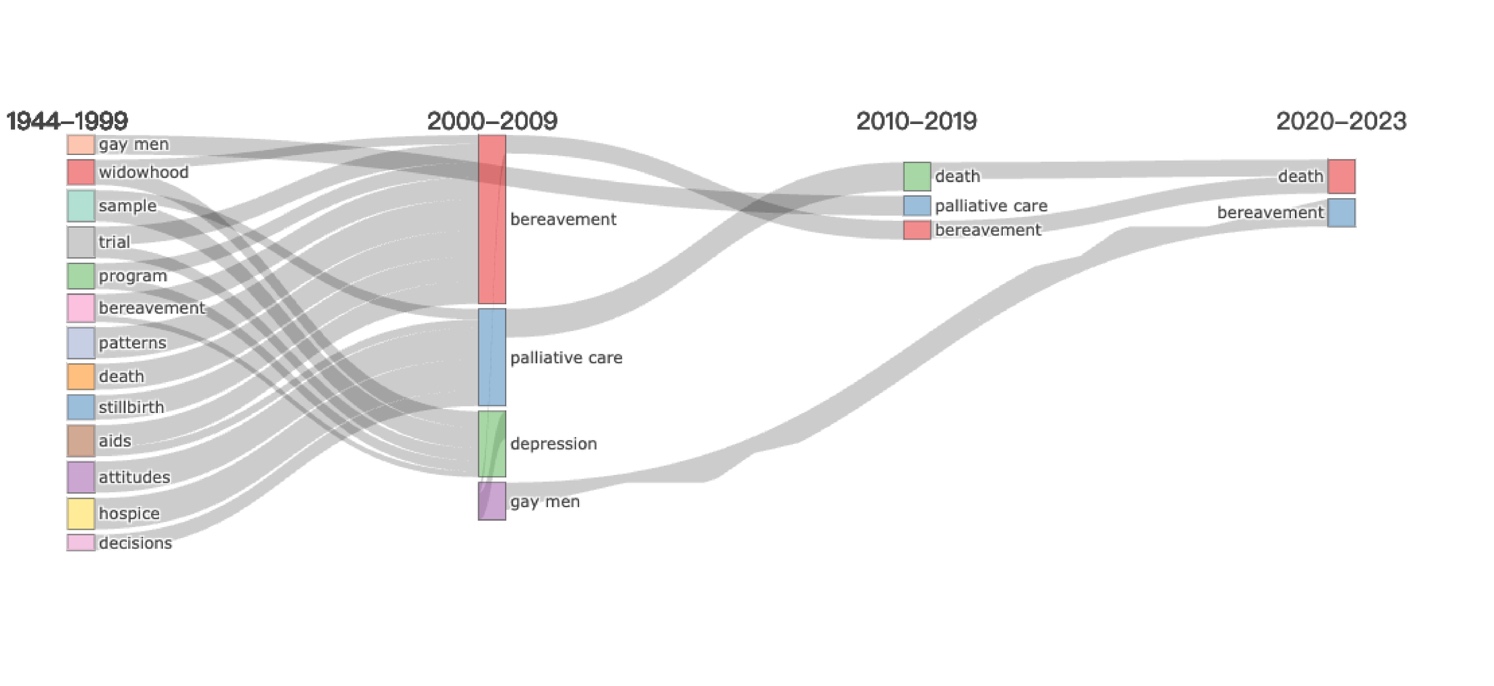


**Supplementary Figure 1.** Thematic evolution across the four-time slices: 1944-2023.


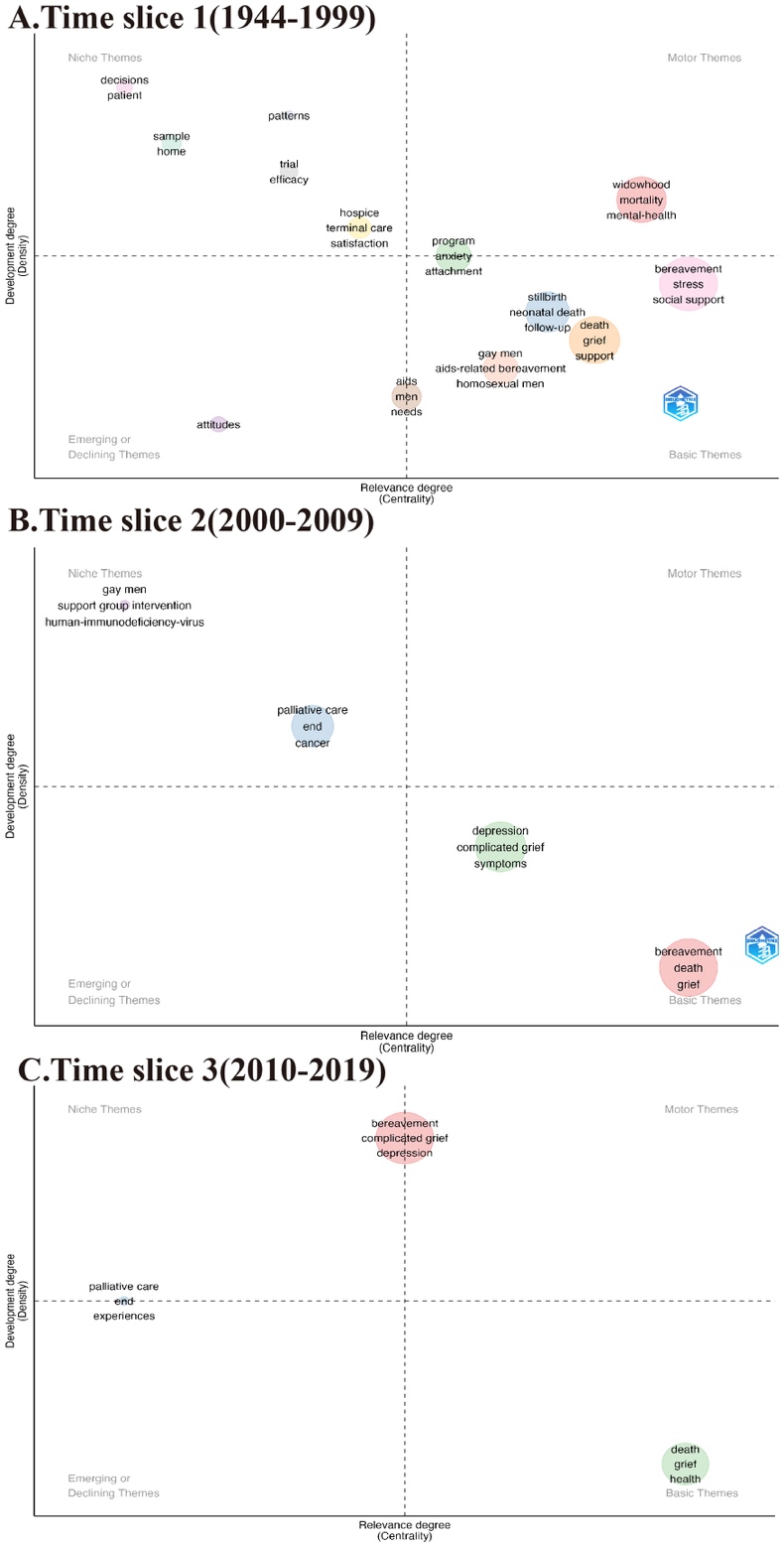


**Supplementary Figure 2.** Thematic maps:(A) research themes in time slice 1; (B) research themes in time slice 2; (C)research themes in time slice 3.(Basic Theme: the fundamental and enduring topics in a research field that have been extensively studied and remain relevant over time. Motor Theme: the current and popular research directions that are attracting significant attention. Niche Theme: the specific and relatively less explored topics that are of interest to a limited group of researchers. Emerging/Declining Theme: the newly emerging or declining topics, indicating a shift in research interes)
